# A Cross-Sectional Study Investigating Cystic Hydatidosis in Slaughtered Cattle of Western Province in Zambia

**DOI:** 10.5402/2013/468163

**Published:** 2012-10-24

**Authors:** Fredrick Banda, King Shimumbo Nalubamba, John Bwalya Muma, Musso Munyeme, Hetron Mweemba Munang'andu

**Affiliations:** ^1^Provincial Veterinary Office, Ministry of Livestock and Fisheries, P.O. Box 910034, Mongu 10106, Zambia; ^2^Department of Clinical Studies, School of Veterinary Medicine, University of Zambia, P.O. Box 32379, Lusaka 10101, Zambia; ^3^Department of Disease Control, School of Veterinary Medicine, University of Zambia, P.O. Box 32379, Lusaka 10101, Zambia; ^4^Section of Aquatic Medicine and Nutrition, Department of Basic Sciences and Aquatic Medicine, Norwegian School of Veterinary Sciences, Ullevålsveien 72, P.O Box 8146 Dep, 0033 Oslo, Norway

## Abstract

A cross-sectional study was conducted from October 2007 to November 2008 to estimate the prevalence of hydatidosis in slaughtered cattle from two abattoirs in Mongu, Western Province, Zambia, using prospective and retrospective data. Out of the 4061 cattle examined during postmortem inspection, 84 (2.1%) were positive for hydatidosis. No cases were detected from Kaoma and Shangombo districts; however, prevalence ranged from 0.6% to 2.5% in districts where it was present. Sex was found to be positively associated with hydatidosis (*P* = 0.035) with female cattle being more likely to have hydatidosis (OR = 1.62). In the retrospective study (1994 to 2007), annual prevalence of hydatidosis ranged from 1.56% (*n* = 12,641) in 2006 to 4.7% (*n* = 2633) in 2001 with an overall prevalence of 3% (4689/158,456). This value is comparable to that observed in cattle slaughtered between October 2007 and November 2008 (2.1%). Hydatidosis was observed in the lungs (51.2%), liver (47.6%), and kidneys (1.2%). The percentage of viable cysts was 43.7%. This study confirms the presence of hydatidosis in cattle in Western Province of Zambia and estimates economic losses due to organ condemnations. Data presented herein provides a useful baseline for developing policy and intervention measures.

## 1. Introduction

Cystic echinococcosis (CE) is a severe zoonosis caused by the cyclophyllidean cestode *Echinococcus granulosus.* The disease has a worldwide distribution, with endemic regions in many countries of the Mediterranean basin, North and East Africa, Western and Central Asia, China, South America, and Australia [1, 2]. Although the distribution of *Echinococcus granulosus *is considered worldwide, it is higher in developing countries in tropics and subtropics, especially in rural communities where there is close contact between dogs and various domestic animals [3]. In some western countries, CE is considered as a reemerging zoonosis due to its resurging prevalence [4, 5]. The worldwide distribution of the disease is partly due to the easy adaptability of the parasite to several domestic and wild intermediate hosts [6]. Clinically, there are three broad morphological forms of echinococcosis that are recognized: cystic echinococcosis caused by *E. granulosus,* alveolar echinococcosis caused by *E. multilocularis,* and polycystic echinococcosis caused by *E. vogeli* and *E. oligathrus* [7–9]. The “sheep” strain (defined as G l on mitochondrial genotypic grounds) is generally considered as the most widespread strain of *E. granulosus* in the world and the one mainly involved in CE in humans [10]. At least five out of ten strains of *E. granulosus* strains (G 1 to G 10) have been found to be infective to humans in sub-Saharan Africa [11].

Echinococcosis has a considerable socioeconomic impact in different countries [12]. In humans, disease consequences may include poor quality of life (disability adjusted life years (DALYS)), costs of medical treatment, lost opportunity for income generation, and mortality in some cases [13] while in animals there is reduced productivity and monetary losses due to abattoir condemnations of organs [14, 15]. The DALYS for human cystic echinococcosis was recently estimated to be more than that for onchocerciasis and almost the same as that for Africa trypanosomiasis [13]. The annual CE-associated economic losses on a global basis have been recently estimated to be at least over US$2 billion [13].

In Zambia, like in most sub-Saharan Africa, echinococcosis has been reported in many parts of the country, although not much information is currently available making it one of the neglected tropical diseases [11]. In Western Province of Zambia, hydatid cysts are reported to have been diagnosed from cattle carcasses during meat inspection although most of these reports are inconclusive [16]. However, there has been no comprehensive study carried out thus far to describe echinococcosis infections in both the intermediate and final hosts and also to determine the economic and public health significance. Based on circumstantial evidence, it is assumed that the disease has serious public health and socioeconomic implications given the interactions that exist between cattle, dogs, and humans and also the uncontrolled disposal of abattoir waste and remains from animal slaughters. However, this assertion needed to be supported by well-structured studies. The aim of this study therefore was to determine the prevalence of hydatidosis in cattle presented for slaughter at abattoirs in Western Province of Zambia and assess economic losses due to organ condemnation using a cross-sectional epidemiological survey with the view to identifying intervention measures aimed at reducing transmission of the disease between humans and different animals hosts.

## 2. Materials and Methods

### 2.1. Study Area

**Figure 1:**
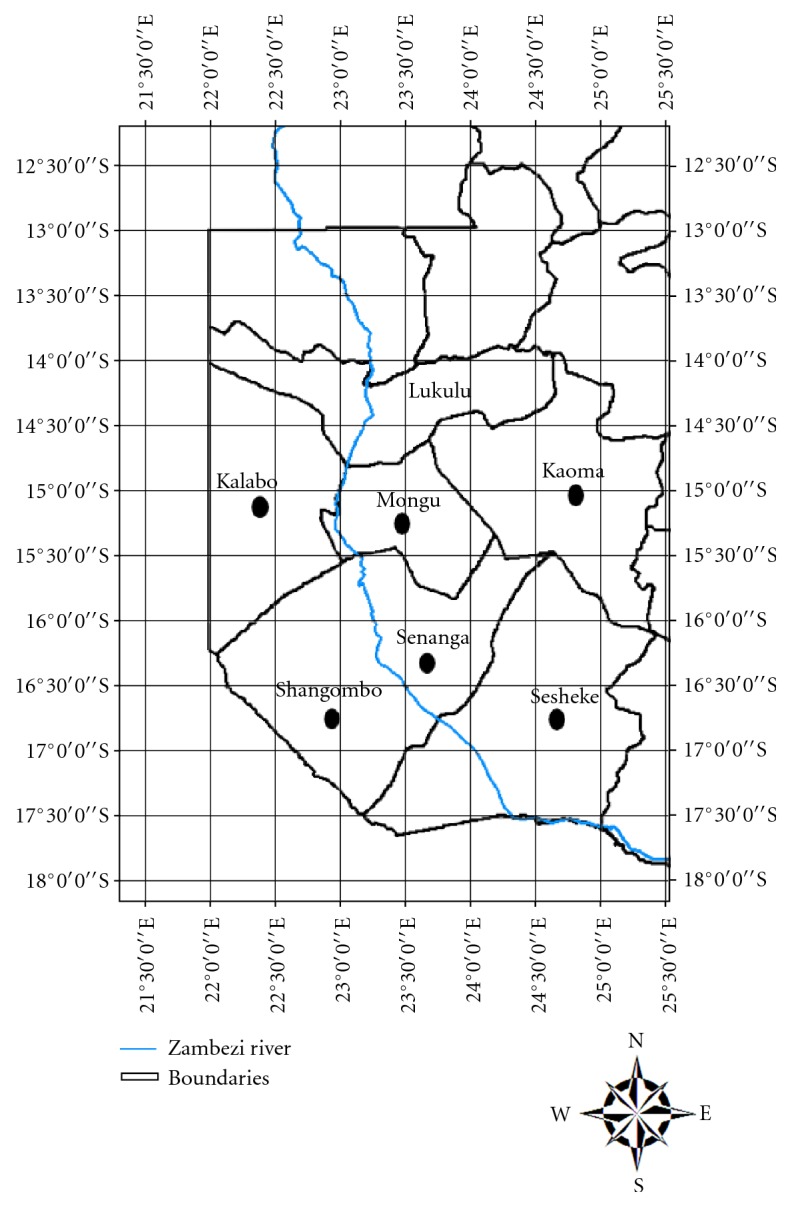
Map of Western Zambia showing the study districts.

The study was conducted in Western Province of Zambia from October 2007 to November 2008. This area was selected because previous reports have suggested that *Echinococcus* spp. infections could be a problem in both cattle and humans. Western Province lies between longitudes 22 degrees and 25 degrees East and latitude 13 degrees 30 mins and 17 degrees 45 mins South. The province covers an area of 126,386 Km^2^, which represents about 17% of the total land surface of Zambia which covers 752,000 Km^2^ (Figure 1). About 10% (12,950 Km^2^) of the total land area consists of a vast sandy upland. The province has a dry and cold winters (April to July), hot and dry season (August to October), and hot and wet summers (November to March). The annual flooding of the Zambezi plains controls the pattern of life for the people and livestock in Western Province with people practice transhumant subsistence livelihood. Thus, during flooding, the largest population of cattle and people are concentrated along the edges of the plains. 

Western Province has a cattle population of approximately 452,400, making it one of the largest cattle producing areas in Zambia, while the dog population is estimated at 65,315 with Mongu having highest number of dogs at 16,210 followed by Kalabo (13,496), Shangombo (11,732), Sesheke (8,638), Kaoma (6,254), Senanga (4,750), and Lukulu (4,236). Dogs, generally, belong to specific households where feeding is supplemented but often have the freedom to roam and scavenge in the neighbourhood. All cattle are slaughtered within the province, mostly in Mongu and Senanga abattoirs, due to a movement ban imposed in 1998 as a result of the outbreak of contagious bovine pleural pneumonia (CBPP). Therefore, data obtained from cattle that are slaughtered in Mongu is a good representation of the true provincial picture.

### 2.2. Study Design

The study was conducted as a two-tier study involving a prospective abattoir survey and a retrospective review of meat inspection reports at Zambeef and Starbeef abattoirs in Mongu.

### 2.3. Retrospective Cattle Study

A retrospective study was carried out based on a review of postmortem reports findings during meat inspection at the abattoirs in the last eleven years (1994–2007). Data was obtained from District Veterinary Offices and abattoir reports on meat inspection and movement of livestock carried out in the previous 11 years in Western Province. Information collected included number of cattle slaughtered, breed and type of organs condemned, number and weight of condemned organs. The aim of this was to provide baseline information and a retrospective understanding of the prevalence, dynamics and spatial distribution of the disease in Western Province and also to estimate the annual economic loss due to organ condemnation.

### 2.4. Prospective Slaughterhouse Survey

This study was conducted between October 2007 and October 2008 at Zambeef and Starbeef abattoirs in Mongu district. Cattle that were slaughtered at the two abattoirs were sourced from all the seven districts of Western Province except Sesheke district. All 4061 cattle that were slaughtered during the study period were included in the survey. An individual animal was considered to be a study unit. The slaughtered cattle were subjected to thorough postmortem inspection and lesioned organs were identified and samples were collected. Prior to commencement of the prospective study, meat inspectors at the two slaughterhouses underwent an in-house refresher training in recognition of hydatid cysts in various organs of the carcasses according to the procedures recommended by FAO/UNEP/WHO (1994). Each animal that was slaughtered was uniquely identified using stock movement permits that included the veterinary camp of origin in the district and further information was obtained by interviewing the owner. The age of the animals was obtained by interviewing the owners in cases where the ultimate owner brought the cattle; otherwise the age was estimated using dentition as described by Jenkins [1].

Visceral organs including lungs, liver, heart, spleen, and kidneys were examined, through visual observation, palpation and systematic incision in each carcass according to procedures recommended by FAO/UNEP/WHO (1994). Hydatid cysts where identified through visual inspection and palpation of organs during meat inspection and enumerated. A sample of hydatid cysts during inspection was removed whole and collected in polythene bags. A separate polythene bag was used for hydatid cysts obtained from one animal and was uniquely labelled and stored in ice before transportation, within one hour, to Mongu regional laboratory for viability determination. Cattle were classified as positive for hydatidosis if it was found with one or more hydatid cysts in any of the internal organs. 

### 2.5. Examination of Cysts and Viability of Protoscolices

At Mongu regional laboratory, the collected cysts were individually grossly examined for degeneration and calcification as described by Oostburg et al. [17]. The cyst wall was carefully incised with a scalpel blade and the contents poured into a Petri dish. The contents were examined under a microscope (40x magnification) for the presence of protoscoleces. The germinal layer was also put in glycerine and placed between two microscopic glass slides and examined for the presence of protoscoleces. Cysts that did not contain protoscoleces contained pus or were calcified were considered as sterile or not viable. Further, the viability of the protoscolices was checked under the microscope using the dye exclusion principle after staining with 0.1% eosin stain for 15 minutes. The protoscolices that took up the stain were classified as dead while those that did not were considered to be alive and viable [18, 19].

### 2.6. Economic Loss Estimation

The loss attributed to condemnations of offal due to echinococcus was determined using a modification of the formula as described by Yamene (1990) cited by Getaw et al. [20]. This was on the basis of the average price of wholesome and intact visceral organs obtained from Zambeef and Starbeef abattoirs Mongu.

### 2.7. Data Handling and Statistical Analysis

Data was stored in Microsoft Excel spread and transferred to Stata statistical packages version 10 (Stata Corp. College Station, TX, USA) for analysis. Significant differences were defined as those with *P* ≤ 0.05. Prevalence of* Echinococcus* spp. infections in cattle for both prospective and retrospective data was determined as proportion of the test-positive subjects against the total number tested. Apparent prevalence estimates were converted into true prevalence values by taking into account the sensitivity and specificity of the test methods as described in Dohoo (2003). Comparisons in prevalences were made between districts and between veterinary camps within each district.

The annual economic loss as a result of condemned organ was estimated by taking into account the average number of cattle slaughtered per annum at the Zambeef and Starbeef abattoir and the percentage of condemned organs using the following formula described by Yemane (1990) as cited by Getaw et al. [20].

(1) Annual  loss=(Nps×Ilu×Clu)+(Nps×Ili×Cli)+(Nps×Ihe×Che)+(Nps×Iki×Cki),

where *N*_ps_: total number of positive animal slaughter, *I*_lu_: prevalence of lung hydatidosis, *I*_li_: prevalence of liver hydatidosis, *I*_he_: prevalence of heart hydatidosis, *I*_ki_: prevalence of kidney hydatidosis, *C*_lu_: cost of lung, *C*_li_: cost of liver, *C*_he_: cost of heart, and *C*_ki_: cost of kidney.

## 3. Results

### 3.1. Retrospective Cattle Study

A retrospective study was carried out based on a review of postmortem report findings during meat inspection at the abattoirs over a period of eleven years from 1994 to 2007 (with exemption of 1997, 1998, and 2002 where data was missing). During this period, 158, 456 bovines were slaughtered and inspected, and 4689 cases of bovine hydatidosis were recorded (Table 2).

The overall combined prevalence of bovine echinococcosis during the period under review was estimated at 3.0% (Table 1) which was close to prevalence observed in our prospective study. Annual prevalence ranged from the lowest at 1.56% (*n* = 12641) in 2006 to the highest at 4.7% (*n* = 2633) in 2001. A review of the postmortem records over an eleven year period revealed that the distribution of hydatid cysts in bovine was highest in lung at 93.47% (95% CI: 92.75–94.14) followed by liver at 6.55% (95% CI: 5.88–7.29) and spleen with the lowest at 0.02% (95% CI: 0.00–0.12) prevalence.

### 3.2. Prevalence of Bovine Echinococcosis from Prospective Surveys

A total of 4061 cattle from Mongu (*n* = 2441), Senanga (*n* = 577), Kalabo (*n* = 653), Lukulu (*n* = 335), Shangombo (*n* = 47), and Kaoma (*n* = 8) were slaughtered at the Zambeef and Starbeef abattoirs between October 2007 and November 2008. Out of this, 84 (2.1%) carcasses (Table 3) were diagnosed positive for hydatidosis during postmortem inspections. There was variation in prevalence of hydatidosis according to the district of origin, where cattle coming from Mongu had the highest prevalence of cyst positive cases (2.5%) compared to Senanga (2.1%), Kalabo (1.4%), and Lukulu (0.6%) (Table 3).

Sex was found to be positively associated with hydatidosis (*P* = 0.035) with female cattle being more likely to test positive than males (Odds ratio = 1.62). On the other hand, hydatidosis was independent of age (*P* = 0.31) where the mean age among the positives was 7.8 years (range: 7.4–7.6) and that among the negatives was 7.5 years (range: 7.3–8.3).

In terms of distribution of hydatid cysts by organ, 51.2% were found in lungs, 47.6% were in livers, while 1.2% were in the kidneys.

Prevalence of bovine hydatidosis was observed to vary according to veterinary camps within districts. Mukukutu camp in Senanga district accounted for the highest prevalence at 4.0% (95% CI 3.8–11.8%), while Lukulu Central camp in Lukulu district had the lowest prevalence at 0.3% (95% CI 0.2–0.9%).

On comparison of camps in different districts, it was observed that in Mongu, the highest prevalence of bovine hydatidosis was in Limulunga veterinary camp at 2.9% (95% CI 1.4–4.4%) with the lowest prevalence in Luandui camp at 1.5% (95% CI 0.5–3.7%). In Senanga district, the highest prevalence was in Mukukutu camp at 4.0% (95% CI 3.8–11.8%) and the lowest was Mouyo camp at 1.6% (95% CI 0.04–3.2%). In Kalabo district, the highest prevalence was observed in Sikongo camp at 3.3% (95% CI 1.2–8.0%). In Lukulu district, the highest prevalence was in Mbanga camp at 1.8% (95% CI 1.7–5.3%) and lowest in Lukulu central camp at 0.3% (95% CI 0.2–0.9%).

### 3.3. Density and Viability of Hydatid Cysts

The overall median number of cysts in an organ was 6 (range 2–21), in the lungs the median was 6 (range 2–21), and liver the median was 4 (range; 3–5). The number of hydatid cysts that were examined in the lung was 108 while in the liver it was 16. The lung had a highest density of cysts per organ compared to the liver (Table 4). There was no significant difference in viability rate of hydatid cysts recovered from the lung (43.5%) and in liver (43.8%).

### 3.4. Evaluation of Economical Losses

The prices used in the estimation of annual economic loss from condemned organs, were the 2011 average prices for wholesome and intact visceral organs obtained from Zambeef butchery in Mongu. While the average weights of the various organs were calculated from the data obtained from the abattoir prospective study. The average weight of a lung was estimated at 2.92 Kg, liver and spleen were 3.34 Kg and 2.00 Kg, respectively.

The average cost of lung was ZMK (Zambian Kwacha) 12,000 per Kg, liver ZMK 18,000 per Kg, and spleen ZMK 12,000. The cost of one lung = average weight × cost/kg (2.92 @ 12000) = ZMK 35,040; cost of liver = average weight × cost/kg (3.34 @ 18000) = ZMK 60,120; cost of spleen= average weight × cost/kg (2 @ 12000) = ZMK24, 000, average annual slaughter= 14,405.

(2) Annual  loss=(14,405×0.027×35,040)+(14,405×0.002×60,120)+(14,405  ×0.001×24,000).

Annual loss = ZMK 15, 894,039 (U$ 3,311). Average exchange rate during the study was 1US$= ZMK 4800. (Source Bank of Zambia, December 2010).

## 4. Discussion

In this study, we investigated the prevalence of hydatidosis based on PM findings at two abattoirs in Western Province of Zambia. It is therefore noted that the prevalence estimates provided here may have some bias as abattoir sample populations is not always representative of the reference populations where animals are drawn. This is often so because animals brought for slaughters are those that are old or out of production. Considering the reduced sensitivity of PM inspection-based diagnosis, there is always a possibility that some positive cases were missed resulting in underreporting the actual disease burden. Despite these short-comings, abattoir survey data is routinely used to estimate disease burden because of easy feasibility of conducting abattoir surveys compared to field surveys based on random study designs. Besides, abattoir data provides opportunity for developing intervention strategies by timely diagnosis and condemning carcasses infected with zoonoses likely to enter the food chain.

The observed prevalence of hydatid cysts in cattle sampled at the two abattoirs in Mongu was found to be low (2.1%) and was comparable to that observed during the retrospective survey (3.0%). Furthermore, the findings in this study were in agreement with that observed in a study done in Sudan which reported a prevalence of 3% in cattle [21]. In Arusha Tanzania, a study by Nonga and Karimuribo reported a prevalence of 4.2% in cattle [22]. Similarly, low levels of bovine hydatidosis were reported by Njoroge in Kenya [23]. In contrast to this, studies done elsewhere in Africa have reported higher prevalence. For instance, Rkia Azlaf and Allal Dakkak reported prevalence of 23.0% bovine hydatidosis in Morocco [12] and so did Kebede in Ethiopia who reported a prevalence of 22.1% [24].

In our study, the distribution of hydatidosis varied according to district with Mongu reporting the highest prevalence compared to other districts. The reason for the high prevalence in Mongu could be attributed to a high numbers of cattle and dog populations coupled with a high number of home slaughters during ceremonies, which in some cases are not inspected by the veterinary department staff [25]. There is an increased dog and cattle interaction due to high populations and free range rearing of cattle which are often herded by boys with dogs; this increases contact of cattle with dog faeces. Further, dog access to slaughterhouse waste in Mongu abattoirs is likely to increase exposure of both cattle and dogs in the district.

Sex was found to be positively associated with hydatidosis (*P* = 0.035) with female cattle being more likely to test positive than males. This is in agreement with the finding by Daryani et al., in Iran, who observed that the prevalence was higher in females than males [26].

There was a significant difference in the prevalence of hydatid cysts between carcasses slaughtered in 2007 and 2008 (*P* = 0.024), where prevalence of bovine hydatidosis in 2007 was 1.3% (95% CI: 0.69–1.93) and in 2008 was 2.4% (95% CI: 1.8–2.9). This could be as a result of more animals coming from areas of higher prevalence of bovine hydatidosis such as Mongu and Senanga in 2008 than in 2007. However, this could not be fully ascertained due to absence of trace back information during the period under review.

The lung was found to be the most affected organ (51.2%) compared to the liver (47.6%) and the kidney (1.2%). This is in agreement with what was reported by Getaw et al. [20] who observed that the lung had a higher prevalence at 55.2% and the liver at 37.1% while the kidney was the least affacted organ [20]. The results are also in agreement with findings by Cadmus and Adesokan (2009) in Nigeria, and Kebede et al. [24] who did their study in Ethiopia. However, our results are at odds with the findings from a study conducted in Libya where researchers reported higher prevalence in the liver than in the lung [27] and Al-khalid (1998) cited by Dakkak [25] who showed that in Libya, 75% of the positive bovine hydatidosis cases were in the liver and 37.5% in the lung and 12.5% in the spleen. The reason why the lung and liver are mostly affected could be due to the fact that the lungs and livers are the first capillary beds encountered by migrating echinococcus oncospheres via the portal vein route before any other peripheral organs. The lungs however have a larger capillary bed than any other organs and this could account for the observed higher prevalence than seen in the other organs [20]. In humans, however, the liver is most commonly affected. The explanation to this differences in the predirection sites between cattle and human is beyond the scope of this study.

Cysts viability study revealed that the overall percentage of viable cyst in this study was 43.5% which is comparable to findings by other researchers like Ibrahim [28] who found cyst viability of 47.8% in sheep and 24% in goats. However, different observations were reported by Rinaldi et al. [29], who did not observe any viable cyst from their survey and Berhe [30] who found a lower viability rate of 10.7% in cattle in Tigray region of Ethiopia. The possible reason why no viable cysts were observed by Rinaldi et al. [29] could be due to the differences in immunological responses by different individual hosts or deworming of the animals by use of antihelmintics [26]. 

Out of a total of 19 hydatid cyst infested organs that were investigated (15 lungs and 4 livers) for cyst fertility, viability, and density, it was found that the lung had a higher average density of cysts infestation (7 cysts per lung) (Table 4) while the liver had a low hydatid cyst density (4 cysts per liver). This was however different from findings by Ibrahim [28] in Saudi Arabia who observed that the liver had a higher cyst density. The difference in the cyst density could mainly be attributed to the higher vascularisation of lung tissue compared to liver [24]. The other reason in the difference in cyst density could be as a result of the soft texture of the lung tissue in comparison to liver [20], which has a harder texture thus restricting hydatid cyst development.

The median number of cysts in an organ was 6 (range 1–21). The number of hydatid cysts that were examined in the lung was 108 while in the liver was 16. Most of the dead cysts in the liver were found to be calcified compared to the lung. This is in agreement with findings by Dalimi et al. [18], Kebede et al. [24], and Berhe [30] who reported a higher fertility rate of pulmonary and lower fertility rate in hepatic cysts. This could probably be due to the various metabolic reactions that take place in the liver as compared to lungs. However Ibrahim [28] found a higher fertility rate in liver at 38.8% than in the lung at 25.1% and so did Dalimi et al. [18] who reported a higher fertility rate in the liver than in the lung.

The high percentage of viable cysts indicates that there is a high risk of dog exposure in situations where offal are carelessly disposed of and dogs have access to the infected offal, like the situation is in Western Province of Zambia. This points to a possible intervention area in which dogs should be prevented from ingesting infected with cysts such as the lungs and liver.

In this study, the annual economic loss as a result of condemnation of organs due to bovine hydatidosis was low at K 15, 894,039.00.(3,311 US$) per annum. The loss was found to be low due to the low prevalence of hydatidosis in cattle in Western Province of Zambia. The total annual loss could be greater than the estimated amount bearing in mind that this only took into account direct losses and not indirect losses as a result of weight loss due to CE and other losses such as reduced milk production and reduction in reproduction in cattle.

## 5. Conclusion

The main thrust of the study was to describe the hydatidosis situation in Western Province so as to increase public health awareness, describe the socioeconomic impact, and to recommend possible mitigation measures. The determination of prevalence of echinococcosis in cattle has some practical implications. These observational studies help detect the disease and estimate prevalence and other parameters easily. It should be noted however, that echinococcosis is a disease of multiple hosts and the objective of this study could only be addressed by the application of conventional observational studies.

This study has demonstrated that hydatidosis is an important disease and is endemic in Western Province. The disease also causes considerable economic losses as a result of offal condemnations. Despite the low infection rate demonstrated by the current study, there are certain socioeconomic conditions that are favourable for the existence of CE, and therefore CE still remains one of the most important helminth zoonotic disease hence the need for increased attention in control and prevention of the disease. 

Livestock and dog echinococcosis prevalence studies and surveillance can help map out CE risk landscape profiles that will determine communities at greatest risk to human CE. A molecular analysis of human and animal hydatidosis would be desirable in order to effectively map out epidemiology of the disease and determine the spread of the disease. A specific study of the disease in dogs could also help in knowing the prevalence in the definitive host. Wildlife species have not been shown to harbour *E. granulosus *in Zambia. In view of the extensive livestock/wildlife interface areas in the province, a study in these would also be useful. Furthermore, a study in small ruminants such as sheep and goats may improve epidemiological understanding of the disease in Zambia. With the current study having been done on cattle, a further study is imperative on dogs so that the epidemiological picture is complete. It is also suggested that in areas where there is a presence of large numbers of wildlife definitive hosts such as observed in some parts of Kalabo, Lukulu, and Kaoma districts, increased effort should be made to sample some of the possible wildlife definitive host of *Echinococcus* spp. Effort should be made to ascertain the host specificity of local strains of the parasite in respect to cattle and other domestic animals. To effectively come up with a control program, possible wildlife reservoirs, and the survival of eggs under the local climatic and soil conditions have to be investigated.

## Figures and Tables

**Table 1:** Distribution of hydatid cysts in cattle carcases by area of origin in traditional cattle (*n* = 4061) in Mongu (2007–2009).

Area of origin	No. of cattle sampled	No. of positives	% positive	95% confidence interval
Mongu	2441	65	2.5	1.9–3.1
Senanga	577	8	2.1	0.9–3.2
Kalabo	653	9	1.4	0.5–2.3
Lukulu	335	2	0.6	0.0–1.4
Shangombo	47	—	0	—
Kaoma	8	—	0	—

Overall	4061	84	2.1	1.6–2.5

**Table 2:** Annual abattoir prevalence of hydatid cysts based on postmortem findings in slaughtered cattle.

Year	No. of slaughtered cattle (*n*)	No. of positives	Condemned organs	Combined prevalence (%)
1994	3500	144	Lung (142)	4.11 (95% CI: 3.49–4.83)
			Liver (2)	
1995	3973	102	Lung (99)	2.59 (95% CI: 2.14–3.13)
			Liver (2)	
			Spleen (1)	
1996	791	15	Lung (15)	1.90 (95% CI: 1.11–3.19)
			Liver (0)	
1999	30497	1103	Lung (1035)	3.62 (95% CI: 3.41–3.84)
			Liver (68)	
2000	14268	630	Lung (616)	4.42 (95% CI: 4.09–4.77)
			Liver (14)	
2001	2633	123	Lung (121)	4.67 (95% CI: 3.91–5.56)
			Liver (2)	
2003	36124	997	Lung (953)	2.76 (95% CI: 2.59–2.94)
			Liver (44)	
2004	25604	645	Lungs (539)	2.52 (95% CI: 2.33–2.72)
			Liver (106)	
2005	10893	456	Lungs (433)	4.19 (95% CI: 3.83–4.59)
			Liver (23)	
2006	12641	197	Lung (182)	1.55 (95% CI: 1.35–1.78)
			Liver (15)	
2007	17532	277	Lung (246)	1.58 (95% CI: 1.40–1.78)
			Liver (31)	

Total	158,456	4,689		2.96 (95% CI: 2.88–3.04)

**Table 3:** Results of a prospective abattoir survey of hydatid cysts based on postmortem findings in cattle slaughtered from Mongu abattoirs, 2007 to 2008.

Variable	Category	Organ	No. of positive with hydatid	% positive	Overall prevalence (95% confidence interval)
District∗∗	Mongu [n = 2441]	Lung	29	1.2	2.7 (1.9–3.1)
		Liver	35	1.4	
		Other	1	0.04	
	Senanga [n = 653]	Lung	5	0.8	1.2 (0.9–3.2)
		Liver	3	0.5	
		Other	0	0	
	Kalabo [n = 577]	Lung	8	0.2	0.3 (0.5–2.3)
		Liver	1	0.2	
		Other	0	0	
	Lukulu [n = 335]	Lung	1	0.3	0.6 (0.0–1.4)
		Liver	1	0.3	
		Other	0	0	

Sex	Male	36	2,215	1.6	1.6 (1.1–1.2)
	Female	48	1,845	2.6	2.6 (1.9–3.2)

Body organ	Lung	43	4061	1.1	
	Liver	40	4061	1.0	2.1
	Kidney	1	4061	0.02	—
	Spleen	0	—	—	—

∗∗Shangombo and Kaoma had no positive cases.

**Table 4:** Viability and intensity of hydatid cysts in different body organs of cattle.

Organ	No. of organs examined	Average no. of cysts per organ	Range of no. of cysts	Mean no. of live cysts (95% CI)	Mean of dead cysts (95% CI)	Average % of live cysts	Average % of dead cysts	Average ratio live/dead cyst	Ratio dead/live cyst
Lung	15	7	2–21	3 (2–4)	4 (2–6)	43.0%	57%	0.8	1.3
Liver	4	4	3–5	2 (1-2)	2 (1–3)	43.7%	56.3	0.8	

Overall	19	6	2–21	4 ( 2–4)	3 (2–5)	43.1%	56.9%	0.8	1.3
